# Supplementary material for: Using a Deep Learning Model to Address Interobserver Variability in the Evaluation of Ulcerative Colitis (UC) Severity
Source: J Pers Med. 2023 Nov 8;13(11):1584. doi: 10.3390/jpm13111584 (PMC10672717; doi:10.3390/jpm13111584)
Supplement: Supplementary file 1 [file jpm-13-01584-s001.zip › jpm-2683854-supplementary.pdf]

Supplementary

Table S1. Deep Learning Performance for Expert A.

Model	Accuracy	F1_score	Recall	Precision
MobileNetV3Large	0.8431	0.8352	0.8431	0.8406
EfficientNetB0	0.8235	0.7955	0.8235	0.8588
VGG16	0.8039	0.7996	0.8039	0.7980
ResNet50	0.7843	0.7697	0.7843	0.7740
VGG19	0.7843	0.7362	0.7843	0.8348
Dense Net 121	0.7647	0.7202	0.7647	0.7724
DenseNet201	0.7451	0.7046	0.7451	0.7294
MobileNetV2	0.7059	0.5842	0.7059	0.4983
InceptionV3	0.7059	0.5842	0.7059	0.4983
ResNet152V2	0.7059	0.5842	0.7059	0.4983
ResNet50V2	0.6863	0.6591	0.6863	0.6517
EfficientNetB7	0.6667	0.6632	0.6667	0.6602
Xception	0.6471	0.6005	0.6471	0.5814

Table S2. Deep Learning Performance for Expert B.

Model	Accuracy	F1_score	Recall	Precision
VGG16	0.8039	0.7723	0.8039	0.8269
ResNet50	0.7255	0.6970	0.7255	0.8062
VGG19	0.7059	0.6643	0.7059	0.7290
MobileNetV3Large	0.6863	0.6749	0.6863	0.7586
EfficientNetB7	0.6667	0.6394	0.6667	0.6611
ResNet50V2	0.6275	0.5722	0.6275	0.7105
Xception	0.6078	0.5702	0.6078	0.6349
EfficientNetB0	0.5882	0.5353	0.5882	0.5636
InceptionV3	0.5294	0.4616	0.5294	0.5571
DenseNet121	0.4902	0.3225	0.4902	0.7501
MobileNetV2	0.4902	0.3225	0.4902	0.7501
DenseNet201	0.4902	0.3312	0.4902	0.2501
ResNet152V2	0.3922	0.2842	0.3922	0.6542

Table S3. Deep Learning Performance for Expert C.

Model	Accuracy	F1_score	Recall	Precision
EfficientNetB0	0.8627	0.8613	0.8627	0.8607
VGG16	0.8431	0.8478	0.8431	0.8630
MobileNetV3Large	0.8235	0.8251	0.8235	0.8274
DenseNet121	0.7843	0.7501	0.7843	0.7941

ResNet50	0.7843	0.7821	0.7843	0.7805
Dense Net201	0.7255	0.6736	0.7255	0.6982
MobileNetV2	0.7059	0.5842	0.7059	0.4983
ResNet50V2	0.7059	0.6402	0.7059	0.6577
VGG19	0.6863	0.6916	0.6863	0.6990
InceptionV3	0.6667	0.6675	0.6667	0.6699
EfficientNetB7	0.6275	0.6402	0.6275	0.6641
ResNet152V2	0.6275	0.6395	0.6275	0.6649
Xception	0.4902	0.5128	0.4902	0.5710

Table S4. Deep Learning Performance for Expert D.

Model	Accuracy	F1_score	Recall	Precision
VGG16	0.6863	0.6841	0.6863	0.7083
EfficientNetB0	0.6471	0.5770	0.6471	0.8036
EfficientNetB7	0.6471	0.6280	0.6471	0.6770
MobileNetV3Large	0.6275	0.5681	0.6275	0.7646
ResNet50	0.6275	0.5695	0.6275	0.6577
MobileNetV2	0.5882	0.5217	0.5882	0.6685
VGG19	0.5882	0.5492	0.5882	0.6498
Xception	0.5490	0.5211	0.5490	0.5201
DenseNet121	0.4510	0.3674	0.4510	0.4291
DenseNet201	0.4510	0.3706	0.4510	0.5742
InceptionV3	0.3725	0.2022	0.3725	0.7662
ResNet50V2	0.3725	0.2022	0.3725	0.7662
ResNet152V2	0.3725	0.2022	0.3725	0.7662

Table S5. Deep Learning Performance for Expert E.

Model	Accuracy	F1_score	Recall	Precision
MobileNetV3Large	0.8824	0.8722	0.8824	0.8992
DenseNet 121	0.8431	0.8296	0.8431	0.8497
EfficientNetB0	0.8235	0.8045	0.8235	0.8319
ResNet50	0.8235	0.8217	0.8235	0.8206
VGG16	0.7647	0.7202	0.7647	0.7724
EfficientNetB7	0.7255	0.7017	0.7255	0.7012
VGG19	0.7255	0.7194	0.7255	0.7156
MobileNetV2	0.7059	0.5842	0.7059	0.4983
ResNet50V2	0.7059	0.5842	0.7059	0.4983
